# Partial phenotype conversion and differential trait response to conditions of husbandry in mice

**DOI:** 10.1007/s00360-017-1138-x

**Published:** 2017-12-06

**Authors:** Julia Brenmoehl, Christina Walz, Marion Spitschak, Elisa Wirthgen, Michael Walz, Martina Langhammer, Armin Tuchscherer, Ronald Naumann, Andreas Hoeflich

**Affiliations:** 1 Institute for Genome Biology, Leibniz-Institute for Farm Animal Biology (FBN), Wilhelm-Stahl-Allee 2, 18196 Dummerstorf, Germany; 2 Institute Biometry and Genetics, Leibniz-Institute for Farm Animal Biology (FBN), Wilhelm-Stahl-Allee 2, 18196 Dummerstorf, Germany; 3 Max Planck Institute of Molecular Cell Biology and Genetics, Pfotenhauerstraße 108, 01307 Dresden, Germany

**Keywords:** Semi-barrier husbandry, SPF husbandry, Body weight, Non-inbred mouse line, Standard chow, Phenotype conversion

## Abstract

Functional genome analysis usually is performed on the level of genotype–phenotype interaction. However, phenotypes also depend on the relations between genomes and environment. In our experimental system, we observed differential response to environmental factors defined by different conditions of husbandry in a semi-barrier unit or in a SPF (specific pathogen free) barrier unit, which resulted in partial reversal of phenotypes previously observed under semi-barrier conditions. To provide an update of basic phenotypes in unselected and randomly mated controls (DUC) and long-term selected DUhTP (Dummerstorf high treadmill performance) mice in the SPF facility, we compared growth parameters, reproductive performance, the accretion of muscle and fat mass, physical activity, and running performance as well as food intake in all experimental groups. For selected parameters, the comparative analysis spans more than 30 generations. In DUC mice, under SPF conditions a more than threefold (*P* < 0.0001) higher subcutaneous fat mass, higher muscle mass by about 25% (*P* < 0.0001), but lower epididymal fat mass in DUhTP mice by about 20% (*P* < 0.0001) were observed. In SPF husbandry, body weight increased to a stronger extent in adult DUC mice (≈ 20%; *P* < 0.0001) than in DUhTP mice (≈ 8%; *P* = 0.001). The concentrations of IGF-1 and IGFBPs in the serum as well as the liver weights were similar in all experimental groups, indicating growth effects independent of the somatotropic axis. Under SPF conditions the litter size at birth increased in DUC mice (*P* < 0.001) but not in DUhTP mice. The differential effect of husbandry on body weights at day 21 and concentrations of triglycerides in the serum of our model were due to the different diets used in the semi-barrier and in the SPF facility. Our results demonstrate differential trait response to environmental factors resulting in partial phenotype conversion in our experimental system. The existence of conditional phenotypes as a result of genotype–environment interactions points to the importance of environmental factors in functional genome analysis.

## Introduction

Since 1983 in Dummerstorf a high treadmill performance mouse model (DUhTP) had been established for the analysis of energy metabolism applying long term phenotype selection for high treadmill performance (Dietl et al. 2004). Selection was performed in the absence of running wheels within the home cage to exclude self-training effects within the selection response. The dimension of the selection experiment is remarkable, because selection period took more than 30 years and included up to 100 families per generation. The initial population of the Dummerstorf outbred mouse lines was a systematic crossbred of four inbred and four outbred founder mouse strains starting in the 1970s (Falkenberg et al. 2000). Both the unselected and randomly mated control line (DUC) and the DUhTP originate from this genetic founder pool. Trait selection in DUhTP was based on a singular submaximal endurance exercise test on a treadmill at day 70 of life (Falkenberg et al. 2000). Without previous training, DUhTP mice cover fourfold increased distances if compared to DUC mice (Brenmoehl et al. 2013). Interestingly, subcutaneous adipose tissue of DUhTP mice is characterized by elevated concentrations of metabolic enzymes for lipid synthesis (fatty acid synthase, acetyl-CoA carboxylase) but also for lipid hydrolysis (hormone-sensitive lipase) or oxidation (long-chain acyl dehydrogenase), respectively, as well as elevated levels of mitochondrial proteins arguing for high mitochondrial biogenesis (Brenmoehl et al. 2015). In response to moderate voluntary physical activity over a period of 3 weeks in running wheels, concentrations of inducer of mitochondrial biogenesis are further increased resulting in efficient fat mobilization in DUhTP mice (Brenmoehl et al. 2015). Recently, we identified genetic linkage of fat cell browning and metabolic health, because young male DUhTP mice are characterized by increased tolerance against oral glucose (Brenmoehl et al. 2016). Under semi-barrier conditions, sedentary DUhTP mice are characterized by increased accumulation of body fat compared to DUC mice (Brenmoehl et al. 2015). After hygienic reorganization of both lines from the semi-barrier to a new SPF barrier unit, we observed differential responses for the new environment resulting in the partial reversal of phenotype, which prompted us to compare isolated phenotypes observed in both mouse facilities. Because we used different diets in the semi-barrier and in the SPF facility, we also assessed the effects of both commercial diets on body weight under identical husbandry conditions.

## Materials and methods

### Animals and husbandry

All in vivo experiments were performed in accordance with national and international animal protection guidelines [German Animal Welfare Act (TierSchG)] and were approved by our internal institutional review board. The study was conducted at the mouse animal facility of the Leibniz Institute for Farm Animal Biology in Dummerstorf, Germany. We used a non-inbred mouse line that has been generated by selection over 130 generations for high treadmill performance (DUhTP) (Falkenberg et al. 2000) and the control mice (DUC) that had been generated from the identical base population without phenotype selection (Dietl et al. 2004). Body weights were simultaneously considered from animals between generations 103 and 137 (DUhTP; each *n* > 23) and between 147 and 182 (DUC; each *n* > 87). For both lines, littermates were registered for every generation and standardized to a number of eight pups per female in the semi barrier unit and to ten pups per mother immediately after birth in the SPF unit. Animals were housed in Makrolon-cages Type II (EBECO, Castrop-Rauxel, Germany) in a semi-barrier system like described recently (Brenmoehl et al. 2013). In the SPF barrier unit, the mice were kept in polysulfone cages of 267 × 207 × 140 mm (H-Temp PSU, Type II, Eurostandard Tecniplast, Germany). Hygiene management and health monitoring in SPF husbandry were performed according to the recommendations of the FELASA (Mahler Convenor et al. 2014). In both conditions of mouse husbandry, environmental conditions were defined by a 12-h light–dark cycle (room temperature 22.5 ± 0.2 °C, humidity 40–60%) and the animals had free access to pellet concentration and water. Semi-barrier animals received food #1314 from Altromin (Lage, Germany). Mice in SPF conditions were fed with autoclaved Ssniff^®^ M-Z food (Ssniff-Spezialdiäten GmbH, Soest, Germany; Table 1). For the resettlement of the new SPF mouse facility in Dummerstorf, by vitrification cryopreserved embryos from individual DUC mice were transferred into pseudopregnant Crl:CD1(ICR) fosters in an existing SPF facility (Max Planck Institute of Molecular Cell Biology and Genetics, Dresden, Germany). New generated DUC_SPF_ mice of generation 150 were then imported to the empty SPF facility in Dummerstorf and expanded as the initial base population. After verification of the SPF status in the new barrier unit (Gesellschaft für innovative Mikroökologie mbH, Michendorf, Germany), fresh recovered embryos from DUhTP_SB_ and DUC_SB_ mice were fresh-transferred to pseudopregnant CD1 foster females and DUC_SPF_ mice.

**Table 1:** Composition of used food

	Altromin #1314	Ssniff M-Z
Dry matter	89.0%	88.0%
Crude protein	22.5%	22.0%
Crude fat	5.0%	4.5%
Crude fiber	4.5%	3.9%
Crude ash	6.5%	6.7%
Nitrogen-free extracts	50.5%	50%
Metabolized energy	2988 kcal/kg	3272 kcal/kg
Calories from protein	27%	36%
Calories from fat	13%	11%
Calories from carbohydrates	60%	53%

Voluntary physical activity and food consumption in both mouse lines were recorded at an age of 49–70 days using running wheels (*d* = 33.4 cm; Tecniplast, Hohenpeißenberg, Germany). In the same period, food consumption was monitored in animals of DUhTP (semi-barrier: generation 105; SPF: generation 128 and 135) and DUC mice (semi-barrier: generation 149; SPF: generation 173 and 180). For the examination of body composition of DUhTP and DUC mice, data from generation 93, 95, 105, 118 (DUhTP_SB_), 137,149,162 (DUC_SB_), 121–124 (DUhTP_SPF_) and 166–167 (DUC_SPF_) were used. Body length determination was performed at day 42 with mice of generation 100 (DUhTP_SB_), 144 (DUC_SB_), 137 (DUhTP_SPF_) and 182 (DUC_SPF_). All mice were killed at day 70 of age via decapitation for blood sampling and tissues (liver, Musculus rectus femoris, epididymal fat, and isolated subcutaneous fat (generation 105, 121–124, 149, 167)) were weighted, snap-frozen in liquid nitrogen, and stored at − 70 °C for subsequent analysis. Serum was stored at − 20 °C. For the longitudinal analysis of body mass after weaning, we analyzed animals of generation 134 (DUhTP_SPF_) and 179 (DUC_SPF_). We included 30 (DUC_SPF_) and 15 (DUhTP_SPF_) standardized litters (*n* = 10), respectively. Furthermore, 30 males and 16 females of DUhTP_SPF_ in generation 135 and DUC_SPF_ mice in generation 180 were divided and afterwards kept for two generations as an own subpopulation without selection procedure (marked as generation 135*–137* and 180*–182*, respectively). Mice in the parallel husbandry were kept in individually ventilated cages (GM500 Mouse IVC Green Line, Tecniplast, Germany) and received either “semi-barrier food” from Altromin or “autoclaved SPF-food” from Ssniff and were analyzed for body masses and food consumption.

### Analysis of the IGF system

Serum concentrations of IGF-1 were analyzed using the specific ELISA for mouse and rat IGF-1 (E25, Mediagnost, Reutlingen, Germany) according to the manufacturer’s instruction. The sensitivity of the assay system was < 0.029 ng/ml and the analytical range for recombinant IGF-1 was 0.5–18 ng/ml. The intra- and inter-assay coefficients of variation (CV%) were 4.2–5.9 and 5.3–6.7%, respectively.

Serum IGFBP-2, -3, and -4 were analyzed by quantitative Western ligand blot analysis as described previously (Wirthgen et al. 2016). Briefly, serum samples were diluted 1:20, boiled in sample buffer (312.5 mM Tris at pH 6.8, 50% (w/v) glycerol, 5 mM EDTA, 1% (w/v) SDS, and 0.02% bromophenol blue) for 5 min. Proteins were separated by 12% SDS-PAGE followed by the transfer onto a polyvinylidene fluoride membrane (Millipore, Bedford, USA). The blots were blocked and then incubated with biotin-labeled human IGF-2 (1:500; BioIGF2-10; ibt-systems, Binzwangen, Germany). The IGF-binding proteins were detected by enhanced chemiluminescence using LuminataTM Forte (Millipore, Bedford, USA). Bands were visualized on KODAK Image Station 4000MM (Molecular Imaging Systems, Carestream Health, Inc., New Haven, USA) and quantified using Gelanalyzer2010a software. Signal intensities were corrected for background and quantified using human recombinant standards (IGFBP-2 to -4, R&D Systems, Wiesbaden-Nordenstadt, Germany) diluted in artificial serum matrix (Biopanda, County Down, United Kingdom) as calibrators on each blot. The calculation of the IGFBP concentrations in plasma was performed using GraphPad Prism6 software and corrected for dilution and volume/lane of each sample. The analytical range for each IGFBP was 150–15000 ng/ml.

### Analysis of serum triglycerides

Triglycerides (TG) were assayed in serum samples using a commercial kit (No. LT-TR 0015; Labor & Technik Eberhard Lehmann, Berlin, Germany).

### Statistical analysis

Data analysis was performed using SAS software (Version 9.4 for Windows, SAS Institute Inc., Cary, NC, USA). Descriptive statistics and tests for normality were calculated with the UNIVARIATE procedure of Base SAS software (SAS Institute Inc. 2013. Base SAS^®^ 9.4 Procedures Guide, Second Edition. Cary,NC: SAS Institute Inc.). Data considered as approximately normal were analyzed by ANOVA using the MIXED procedure of SAS/STAT software (SAS Institute Inc. 2013. SAS/STAT^®^ 13.1 User’s Guide. Cary, NC: SAS Institute Inc.). The ANOVA model for the 70d data contained the fixed factors line (levels: DUC, DUhTP), group (levels SB, SPF) and the interaction line × group. Homogeneity of covariance parameters across groups was tested for all ANOVA models. In addition, least-squares means (LSM) and their standard errors (SE) were computed for each fixed effect in the models, and all pairwise differences of LS-means were tested by the Tukey–Kramer procedure. Effects and differences were considered significant if *P* < 0.05.

## Results

### Body mass as a function of animal husbandry

**Fig. 1:**
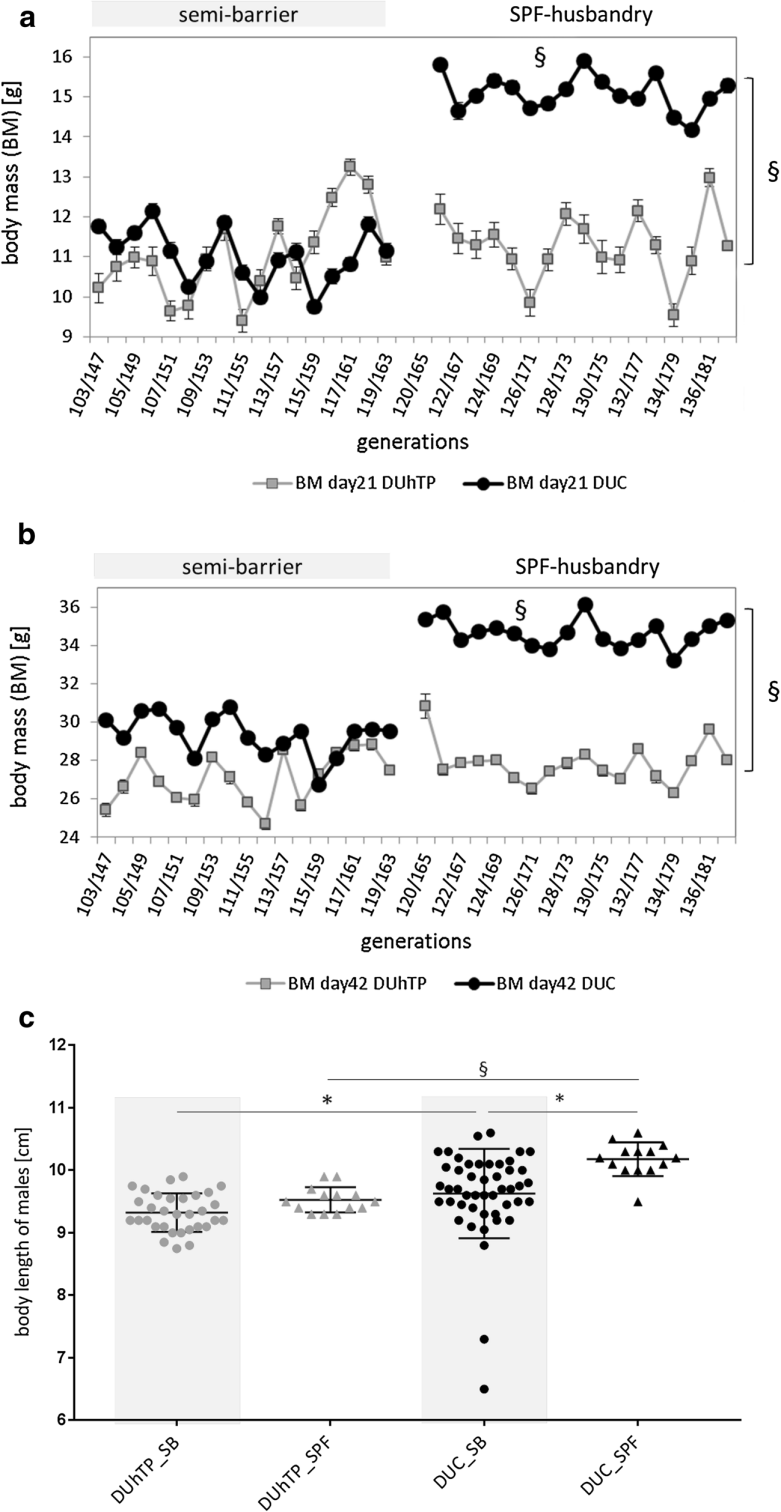
Body mass and body length of male DUhTP (grey data points) and DUC mice (black data points) kept under semi-barrier or SPF conditions. Body mass is presented for an age of 21 days (**a**) and 42 days after birth (**b**). Body lengths were recorded at day 42 in both mouse lines (**c**). Animals were kept in semi-barrier (dots) and SPF husbandry (triangles), respectively. Data are presented as means ± SE (*P < 0.05, § *P* < 0.0001)

The mouse models DUC and DUhTP were established under semi-barrier conditions starting in the early 1970s and 1980s, respectively. In 2012, all mouse lines were transferred from the semi-barrier unit to the newly built mouse facility with standardized environmental and working procedures defined by the SPF status. Longitudinal analysis of body mass in DUC and DUhTP mice revealed positive effects of SPF husbandry only in DUC but not in DUhTP mice (Fig. 1). At an age of 21 days, DUC_SPF_ mice were heavier by 36.4% (*P* < 0.0001) if compared to DUC mice in the semi-barrier (Fig. 1a). Also, at an age of 42 days, DUC_SPF_ mice were characterized by elevated body mass if compared to DUC mice kept in the semi-barrier (+ 18%; *P* < 0.0001; Fig. 1b). By contrast, both conditions of husbandry did not impact on body mass of DUhTP mice at an age of 21 or 42 days. Under SPF conditions, DUC mice also were significantly taller than mice of the same line in SB husbandry (10.18 ± 0.072 versus 9.63 ± 0.104 cm, *P* < 0.01) while the body lengths of DUhTP mice were similar in both facilities (Fig. 1c). Consequently, body lengths differed between both mouse lines in SPF husbandry by more almost 7% (*P* < 0.0001). Under semi-barrier conditions, DUhTP mice were smaller by only 3% (*P* < 0.05).

**Fig. 2:**
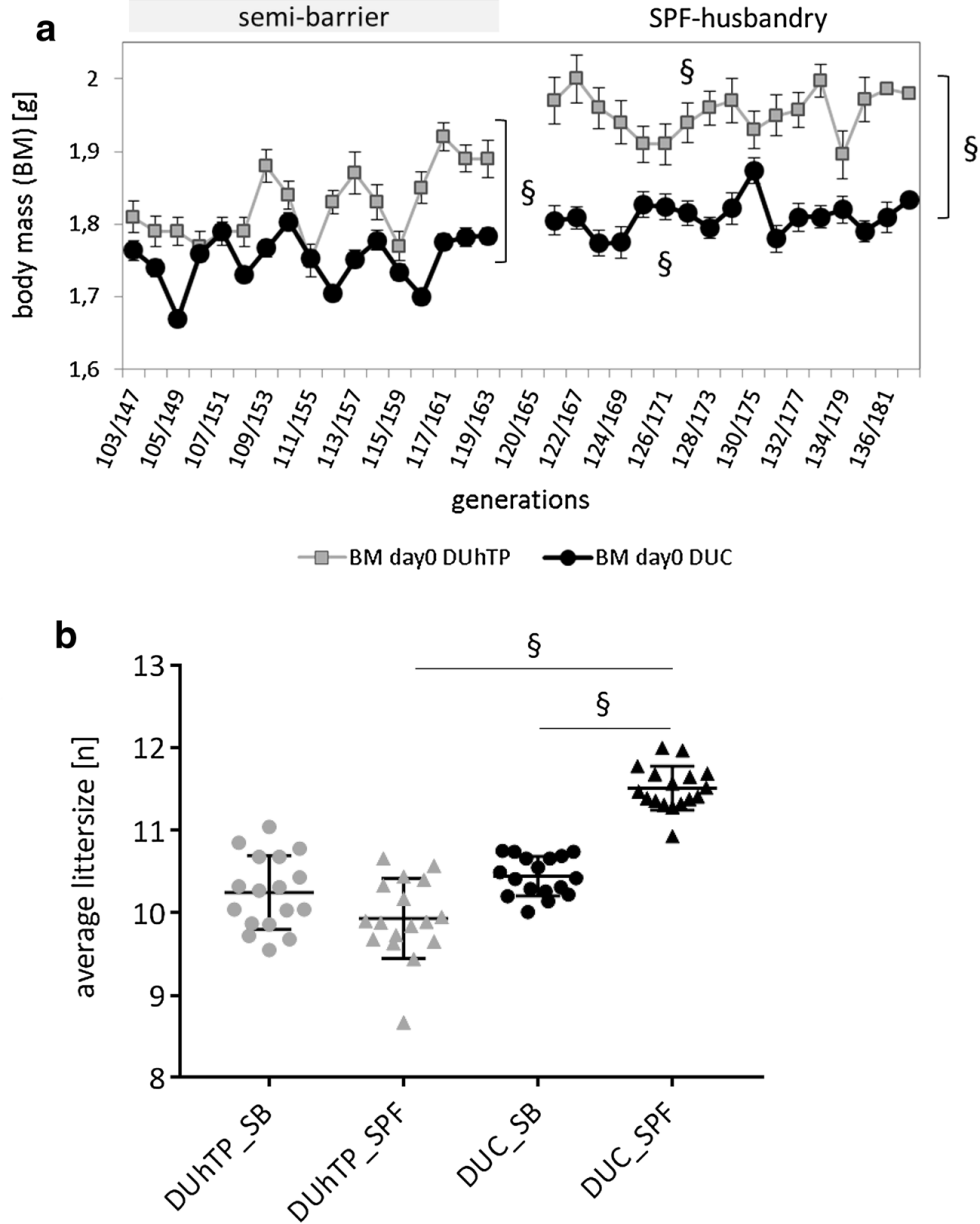
Birth weight (**a**) and litter size (**b**) in DUhTP (grey data points) and DUC mice (black data points) kept under semi-barrier or SPF conditions. Data are presented as means ± SE (§ *P* < 0.0001)

At birth, SPF conditions increased body weights in both mouse lines (DUC + 3.3%; DUhTP + 6.9%; both *P* < 0.0001; Fig. 2a). The body weights of DUhTP mice were higher at birth if compared to DUC mice (BM0 in DUhTP 1.95 g ± 0.03; BM0 in DUC 1.81 g ± 0.02; *P* < 0.0001; Fig. 2a). Interestingly, litter size was significantly increased by SPF husbandry exclusively in DUC mice (DUC + 1.07 littermate, *P* < 0.0001; Fig. 2b), which further increased the difference of litter size between DUC_SPF_ and DUhTP_SPF_ mice (+ 13.7%, *P* < 0.0001).

### Postnatal weight gain

**Fig. 3:**
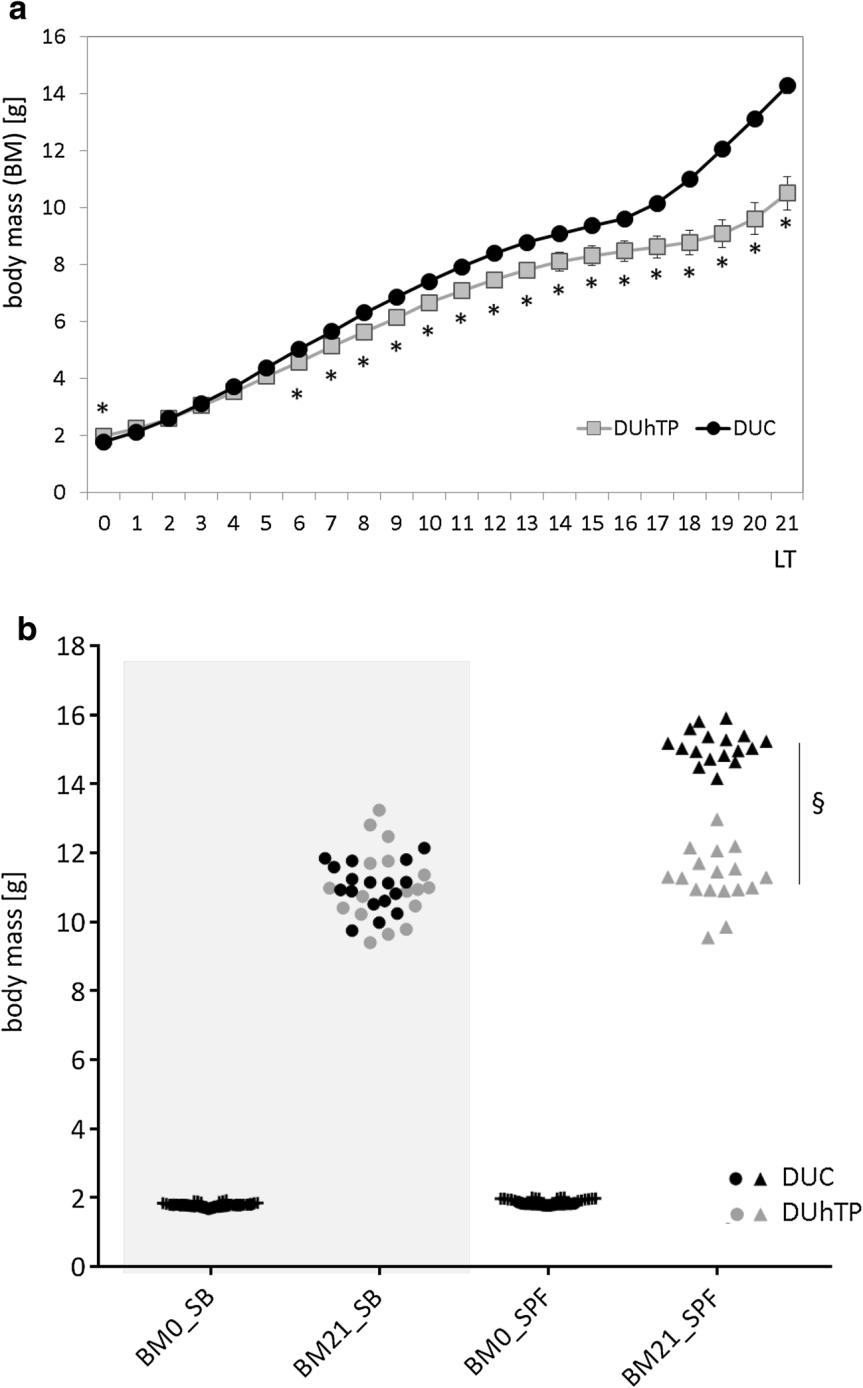
Postnatal weight gain in DUhTP (grey data points) and DUC mice (black data points) kept under SPF conditions (**a**) and effect of animal husbandry on postnatal weight gain (**b**) in both mouse lines. For the comparison of weight gain, animals of individual generations were kept in semi-barrier (dots) and SPF husbandry (triangle), respectively. One data point represents one generation. Body mass of litter size-standardized pups (*n* = 10) between day 0 and 21 after birth was determined in DUC (*n* = 30, generation 134) and DUhTP (*n* = 15, generation 179) littermates. Data are presented as means ± SE (* *P* < 0.05, § *P* < 0.0001)

Longitudinal assessment of body mass in SPF husbandry revealed higher body weights in DUC_SPF_ versus DUhTP_SPF_ mice shortly between days 6 and 21 after birth (Fig. 3a). DUC_SPF_ offspring has significantly increased level of postnatal weight gain immediately after birth prolonging the whole suckling period. In fact, postnatal weight gain from birth to day 21 identified significantly higher mass accretion in DUC_SPF_ offspring if compared to DUhTP_SPF_ mice (Fig. 3b).

### Differential effects of animal husbandry on organ weights

At an age of 70 days under semi-barrier conditions, body and liver weights were higher in DUC_SB_ mice (*P* < 0.01), whereas epididymal and subcutaneous fat depots revealed decrease weights (*P* < 0.001) if compared to DUhTP_SB_ mice (Table 2). Under SPF conditions, higher body and liver weights also were observed in DUC_SPF_ mice (*P* < 0.001). However, the increases of body weights in DUC_SPF_ mice were much higher if compared to semi-barrier husbandry (*P* < 0.0001). In DUC mice, maintained under SPF conditions a more than threefold increase of subcutaneous fat mass was observed if compared to semi-barrier husbandry (*P* < 0.0001). In DUhTP_SPF_, an increase of subcutaneous fat by only 25% was found if kept under SPF conditions (not significant). The increase of epididymal fat by about 30% only has borderline significance (*P* = 0.067). By contrast, in DUhTP_SPF_ mice epididymal fat mass was decreased if kept under SPF conditions (− 30%; *P* < 0.0001). Thus, DUhTP mice, characterized by higher fat mass under semi-barrier conditions (*P* < 0.001), have lower fat mass under SPF conditions (*P* < 0.001) if compared to DUC_SPF_ mice.

**Table 2:** Overview of body masses and tissue weights of 70 day old male DUhTP and DUC mice from individual generations

	*n*	DUhTP_SB	DUhTP_SPF	*P*	DUC_SB	DUC_SPF	*P*	*P* (DUhTP_SB versus DUC_SB)	*P* (DUhTP_SPF versus DUC_SPF)
Body mass (g)	> 24	30.14 ± 0.60	32.61 ± 0.30	**0.00111**	33.93 ± 0.54	40.52 ± 0.63	< **0.00001**	**0.00002**	< **0.00001**
M. rectus femoris (g)	> 24	0.41 ± 0.01	0.38 ± 0.01	0.10401	0.39 ± 0.01	0.49 ± 0.01	< **0.00001**	0.69878	< **0.00001**
Liver mass (g)	> 24	1.73 ± 0.05	1.74 ± 0.02	0.99655	1.96 ± 0.05	1.97 ± 0.04	0.99983	**0.00143**	**0.00084**
Subcutaneous fat (g)	> 10	0.36 ± 0.03	0.45 ± 0.02	0.44078	0.20 ± 0.01	0.68 ± 0.06	< **0.00001**	**0.00003**	**0.00006**
Epidymal fat (g)	> 21	0.43 ± 0.02	0.24 ± 0.01	< **0.00001**	0.29 ± 0.02	0.38 ± 0.03	0.06680	**0.00043**	**0.00004**
Food consumption (g)	> 8	124.52 ± 7.54	116.79 ± 2.91	0.7332	142.01 ± 9.99	127.85 ± 3.01	0.25107	0.22265	0.22284
RW activity (rounds/3 weeks)	> 19	91,760.5 ± 9292.9	85,651.1 ± 6134.6	0.9578	101,535.9 ± 10,343.6	94,520.4 ± 8245.3	0.94397	0.89926	0.8239

Voluntary running wheel activity and food consumption of DUhTP and DUC mice of individual generations were determined from day 49 to 70. Mice were kept in semi-barrier (SB) and SPF condition. All values are means ± SE; *P* values as indicated; significant differences are printed in bold typeface

Generally, while control mice DUC_SB_ in semi-barrier husbandry were characterized by decreased epididymal and subcutaneous fat in contrast to DUhTP_SB_ mice, they show now higher fat masses than DUhTP_SPF_ mice.

**Fig. 4:**
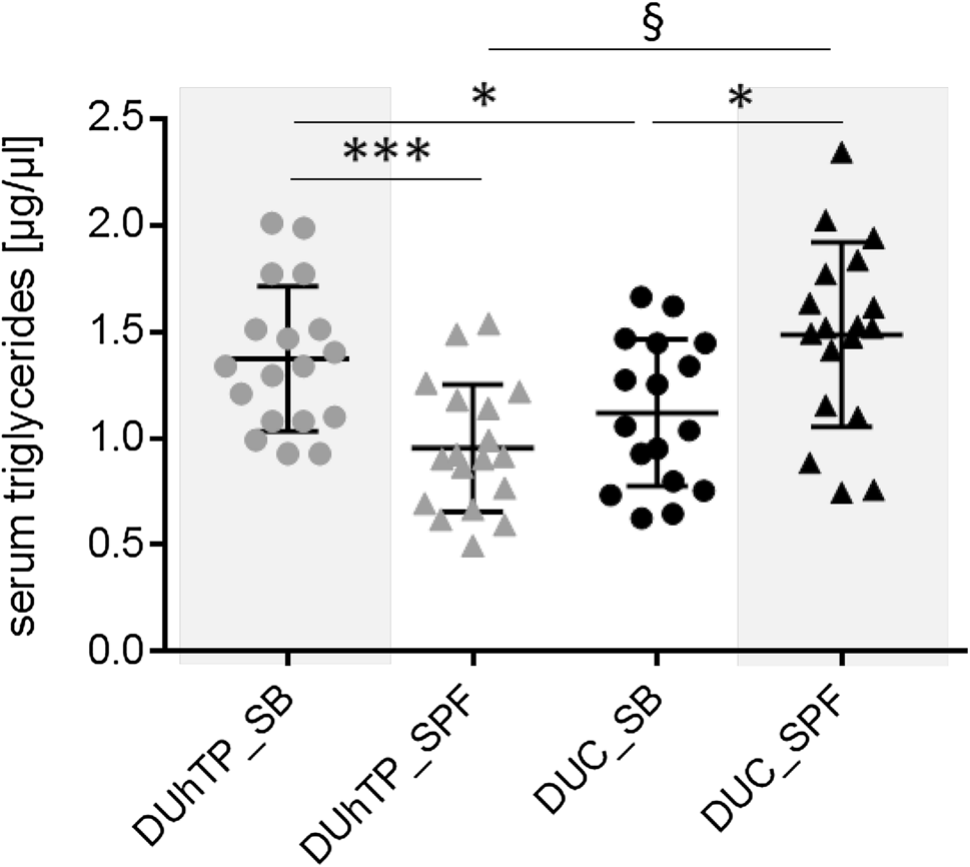
Concentrations of triglycerides in serum of 70-day-old male DUhTP (grey data points) and DUC mice (black data points). Mice were kept in a semi-barrier (SB; dots) or in an SPF facility (triangles). Data are presented as means ± SE (*P < 0.05, ***P < 0.0005, § *P* < 0.0001)

In response to SPF husbandry, concentrations of serum TG were decreased in DUhTP (*P* < 0.0005; Fig. 4) and increased in DUC mice (*P* < 0.05). In SB conditions, DUhTP mice were characterized by higher TG level when compared to controls (*P* < 0.05) while under SPF conditions DUC mice had higher TG concentrations than DUhTP mice (*P* < 0.0001).

### Effects of animal husbandry on the IGF system

With respect to the differential effects of animal husbandry on body weight, we assessed serum concentrations of IGF-1 and IGFBPs in both mouse lines maintained under semi-barrier and SPF conditions. However, no significant differences of IGF-1 or IGFBP-2, -3, and -4 could be observed (Table 3). In all conditions assessed, serum concentrations of IGF-1 and IGFBPs were highly variable in both mouse lines.

**Table 3:** Serum concentrations of IGF-1, IGFBP-3, IGFBP-2, IGFBP-4 and the ratio between IGFBP-3/IGFBP-2 in 70-day-old DUhTP and DUC mice

	*n*	DUhTP_SB	DUhTP_SPF	*P*	DUC_SB	DUC_SPF	*P*	*P* (DUhTP_SB versus DUC_SB)	*P* (DUhTP_SPF versus DUC_SPF)
IGF1 (ng/ml)	> 16	308.66 ± 70.58	168.38 ± 49.91	0.12603	192.92 ± 57.10	268.55 ± 74.75	0.44114	0.22510	0.36204
IGFBP-3 (ng/ml)	> 16	1171.43 ± 248.59	769.28 ± 168.81	0.20437	683.12 ± 141.18	875.99 ± 167.67	0.39937	0.10963	0.68958
IGFBP-2 (ng/ml)	> 16	435.76 ± 79.02	344.10 ± 67.90	0.40100	383.36 ± 68.08	367.25 ± 61.6	0.86575	0.62908	0.70329
IGFBP-4 (ng/ml)	> 16	193.35 ± 30.42	200.62 ± 28.29	0.86884	165.33 ± 24.43	258.49 ± 51.32	0.12853	0.49974	0.40422
IGFBP-3/IGFBP-2	> 16	2.90 ± 0.44	2.44 ± 0.48	0.50454	1.89 ± 0.33	3.61 ± 1.05	0.12490	0.08496	0.37222

Animals were kept in semi-barrier (SB) and SPF husbandry, respectively. All values are means ± SE. *P* values as indicated

### Effects of animal husbandry on running performance, physical activity, and food consumption

**Fig. 5:**
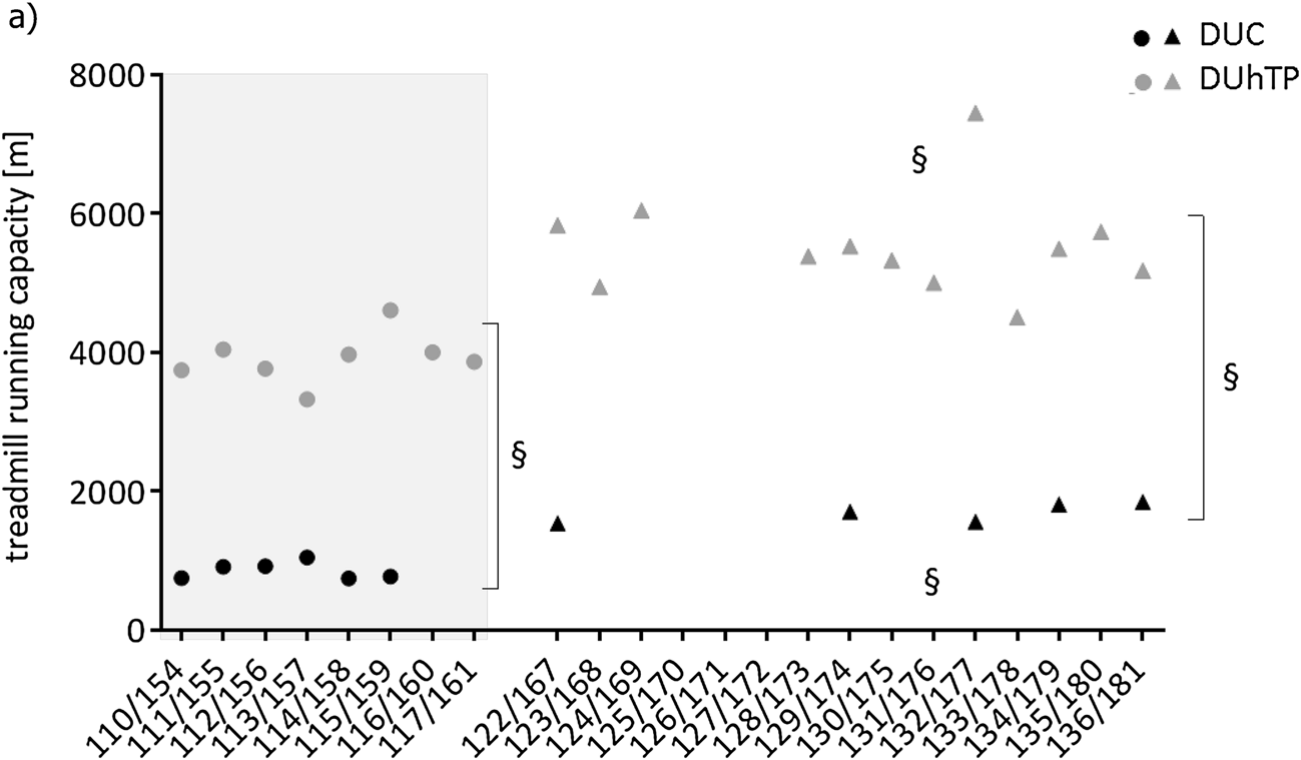
Long-term assessment of submaximal running capacity in male DUhTP (grey) and DUC mice (black) at an age of 70 days. Between generation 110 and 117 for line DUhTP or between generation 154 and 159 for line DUC, respectively, the animals were kept in semi-barrier conditions (dots). Running performance of DUhTP mice after generation 120 or of DUC mice after 165 generations was assessed in mice maintained under SPF conditions (triangles). Data are presented as means ± SE (§ *P* < 0.0001)

SPF husbandry significantly increased submaximal running performance activity in both mouse lines (*P* < 0.0001) if compared to the semi-barrier (Fig. 5). In DUC_SPF_ or DUhTP_SPF_ mice, an almost 100 or 40% increase was observed, respectively. In absolute terms, the increase of submaximal running performance in DUhTP_SPF_ mice was almost twofold if compared to DUC_SPF_ mice (DUhTP_SPF_ + 1620 m; DUC_SPF_ + 836 m). Thus, DUhTP_SPF_ mice were able to maintain their prominent phenotype of higher running capacities if compared to unselected control mice also under conditions of SPF husbandry (*P* < 0.00001). Voluntary physical activity in running wheels and food consumption, examined at an age between 49 and 70 days during semi-barrier and SPF husbandry in both mouse facilities, were similar in all experimental groups (Table 2).

### Effect of food on the body weights

**Fig. 6:**
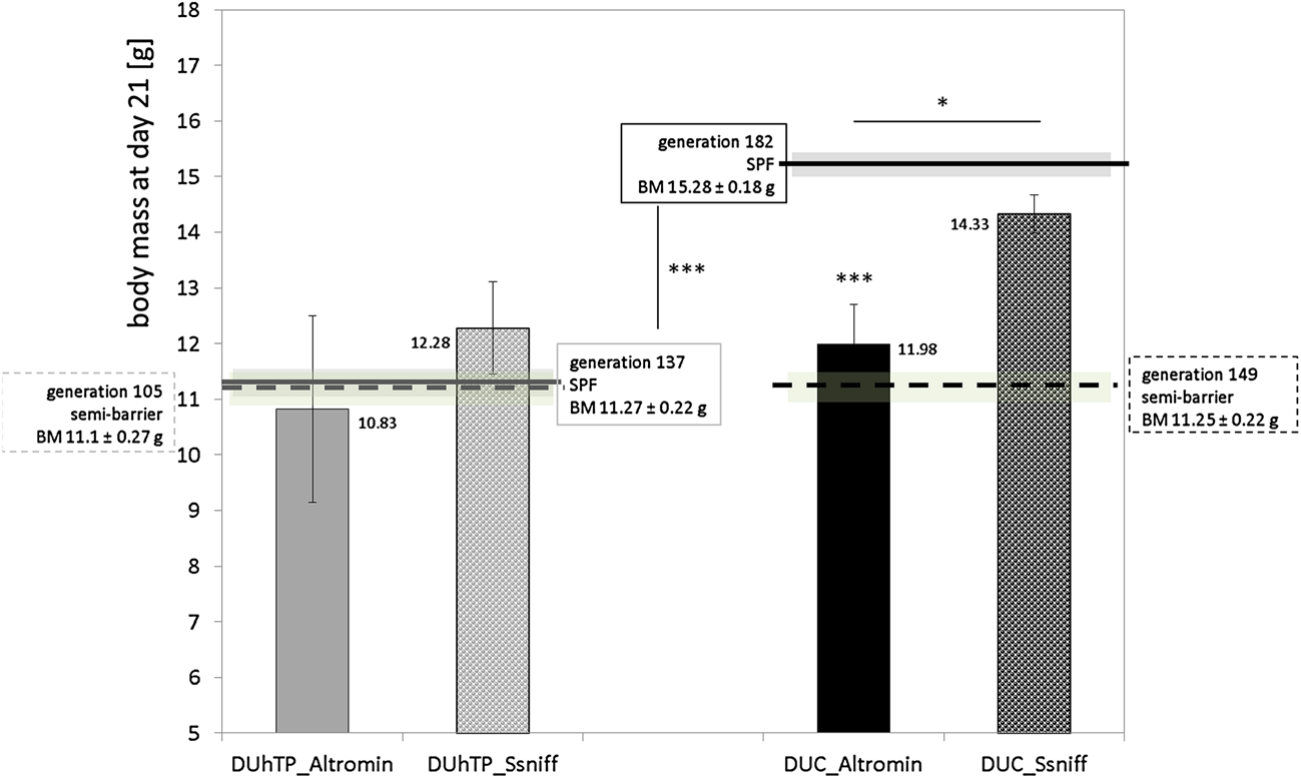
Effect of diet on body mass in male DUhTP mice (grey bars) and unselected controls (DUC; black bars) maintained under SPF conditions at an age of 21 days. For the experiment, mice of both lines were kept in the quarantine station and either received Altromin diet fed in the semi-barrier or autoclaved Ssniff^®^ diet fed in the SPF facility. The dashed line indicates body weights under conditions of semi-barrier husbandry (DUhTP: generation 105; DUC: generation 149). The continuous line displays body weights monitored within the SPF facility (DUhTP: generation 137; DUC: generation 182). Data are presented as means ± SE (*P < 0.05, ***P < 0.0005)

**Fig. 7:**
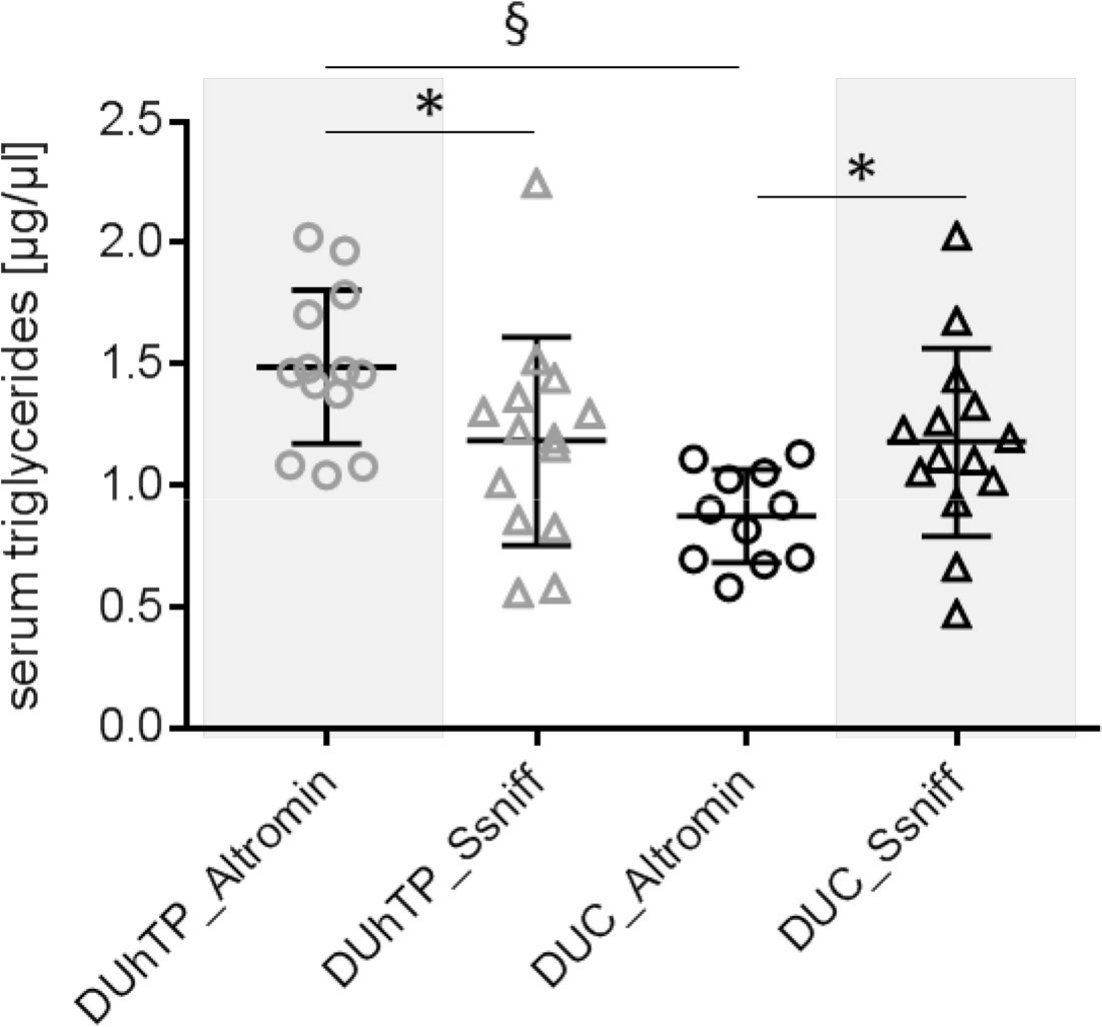
Effect of diet on concentrations of triglycerides in serum obtained from male DUhTP (grey) and DUC mice (black data points) at an age of 70 days. Mice were kept in the quarantine station under SPF conditions and received SB (Altromin, open dots) and autoclaved SPF diet (Ssniff, open triangles), respectively. Data are presented as means ± SE (*P < 0.05, § *P* < 0.0001)

Feeding semi-barrier diet (Altromin) under SPF conditions for two generations in a subpopulation resulted in significantly reduced body weight in DUC_SPF_ mice (*P* < 0.0001) and as a tendency also in DUhTP_SPF_ mice (not significant) at an age of 21 days compared to the Ssniff diet (Fig. 6). While Ssniff-fed DUC mice had a mean body mass of 14.3 g corresponding to the weights of animals in SPF husbandry (15.28 ± 0.18 g), body weights of Altromin-receiving mice were decreased to an average mass of 11.98 g. Therefore, the body weights correspond to those of former animals in semi-barrier husbandry (DUhTP_SB_ 11.25 ± 0.22 g; generation 149). Altromin diet in DUhTP mice increased TG, whereas in DUC mice the same diet reduced the concentrations of TG in serum if compared to autoclaved Ssniff diet (Fig. 7).

## Discussion

The Dummerstorf high treadmill performance mouse model DUhTP established by long-term selection for high treadmill performance is characterized by highly efficient fat mobilization (Brenmoehl et al. 2015), increased fat cell browning, and higher surface temperature (Brenmoehl et al. 2016) if compared to DUC mice deriving from the identical genetic background at the beginning of the selection experiment. Under conditions of Altromin1314 diet in the semi-barrier, DUhTP mice had a significant lower body mass than controls at day 70 but not at day 49 of age (Brenmoehl et al. 2013, 2015). After hygienic reorganization and establishing of Dummerstorf mouse lines in a new building, defined by the SPF status, we observed line-specific effects of the new environment. Due to the huge dimensions of the selection experiment—spanning more than 35 years now—and concerning the non-inbred structure of the genetic background—maintained by inclusion of up to 100 families per litter—the current phenotype of the mouse model requires an update and a discussion of potential effects due to the relocation or SPF husbandry in DUhTP versus DUC mice.

### Body and organ weights

As a major effect of relocation or SPF husbandry, body weights and lengths were increased in DUC_SPF_ mice to a significantly higher dimension if compared to DUhTP_SPF_ mice. The stronger effects of SPF husbandry were evident in mice older than 5 days, when DUC_SPF_ mice have higher body weights due to an increased body weight gain than DUhTP_SPF_ mice. It could be assumed that the higher living weight of the DUC mothers offers a better rearing capacity with a better milk supply for the offspring. At an age of 21 and 42 days, DUC_SPF_ mice have significantly increased body weights also if compared to DUC_SB_ mice maintained under semi-barrier conditions. In addition, SPF husbandry differentially affected the accretion of adipose and muscular tissues in both mouse lines. Accordingly, exclusively in DUC_SPF_ mice, muscle mass as well as the amounts of subcutaneous or epididymal fat were increased by SPF husbandry. By contrast, epididymal fat mass was decreased in DUhTP_SPF_ mice if compared to DUhTP_SB_ mice. The differential effects of animal husbandry on fat mass in both mouse lines were reflected also on the level of serum triglycerides, which were decreased by SPF in DUhTP but increased in DUC mice maintained under SPF conditions. For the increase of body weight in both mouse lines, we thus have to consider dietary factors such as food intake and macronutrient composition (Hall and Guo 2017). More generally, food intake or the input of metabolizable energy were similar in both genetic lines and in both treatment groups at an age of 70 days (DUhTP_SB_ 831.3 ± 89.0 kcal, DUhTP_SPF_ 787.9 ± 42.9 kcal and DUC_SB_ 858.9 ± 36.5 kcal, DUC_SPF_ 812.7 ± 99.8 kcal). However, Ssniff diet contained more metabolizable energy from protein than Altromin (Tabel 2; Ssniff 36%; Altromin 27%). Accordingly, the expense of energy metabolizable from fat (Ssniff 11%; Altromin 13%) or of carbohydrates (Ssniff 53%; Altromin 60%) was higher in Altromin food. In adult mice, the use of high-protein diets can promote weight loss via reduction of fat accretion (Petzke et al. 2014). In the growth period, amino acids have been provided to highly efficient support growth rates in rats (Rogers and Harper 1965). In rats, direct comparison of a high versus low protein diet during pregnancy and lactation revealed higher growth rates in the offspring in a sex-specific manner (Thone-Reineke et al. 2006). The protein-to-energy ratio also was demonstrated in non-rodent animals as an important determinant of weight gain (Garling and Wilson 1976; Dong et al. 2017). An excess of protein intake in mice by means of a 40% protein diet, impaired postnatal growth (Kucia et al. 2011), supporting a major effect of macronutrient composition also in mice. In addition to diet under SPF husbandry, also an improved animal health status due to the absence of defined pathogens has to be considered. To specifically address the effects of Ssniff versus Altromin diet, we applied both diets under SPF conditions over a period of two generations in a subpopulation. In fact, Altromin diet significantly reduced body weight in DUC mice in the absence of specific pathogens under conditions of SPF husbandry. Accordingly, body weights in DUC mice were no longer different if both treatments were compared identifying diet as the causative factor for the prominent weight increases and not the improved health status. In addition, feeding Altromin diet under SPF conditions reversed TG levels in both mouse lines resulting in a relative increase in DUhTP mice but in a decrease in DUC mice. Interestingly, increased protein intake by use of the earlier mentioned high-protein diet impaired liver weights (Kucia et al. 2011) and regulated hepatic expression of the GH/IGF pathway in the perinatal period in mice (Vanselow et al. 2011). In DUC_SPF_ mice, liver weights were unaffected by husbandry in both mouse lines. Because liver is a sensitive target for the effects of growth hormone (GH) (Bates et al. 1964), we may assume that accelerated growth in DUC mice in response to SPF husbandry is not related to altered GH. This assumption is further supported by similar serum concentrations of IGF-1 and IGFBP-3 in all experimental conditions. Both IGF-I and IGFBP-3 represent established biomarkers for GH secretion (Blum et al. 1993). Also, the serum levels of IGFBP-2 were similar in both mouse lines and during semi-barrier or SPF husbandry. Because IGFBP-2 is potently suppressed by GH, IGFBP-2 represents an indirect marker of GH secretion (Kirsch et al. 2007). Finally, the ratio of IGFBP-3/IGFBP-2 as the most sensitive marker of GH secretion (Mesotten 2008) was unaffected by genotype or treatment, which further did not support effects of diet on GH secretion in our experimental system.

The accretion of muscle mass or body fat is related to physical activity or food consumption. However, both parameters also were similar both in DUC mice and in DUhTP mice independent of husbandry.

### Litter size and birth weight

Relocation of DUC and DUhTP mice from the semi-barrier to the SPF facility increased the average litter size in DUC_SPF_ mice but not in DUhTP_SPF_ mice. Litter size is a function of inbreeding (Falconer 1960), and inbreeding negatively affects litter size. Two arguments do not support an effect of inbreeding on the increase of litter size in DUC_SPF_ mice. On one hand, during relocation not all DUC families could be transferred resulting in a reduction of genetic complexity in DUC mice. In addition, the increase in litter size in DUC mice was sudden and appears to be stable, which also may argue against a long-term effect of outbreeding. Due to the high number of founder animals in DUC with 110 and in DUhTP with 46 mice during relocation process in both mouse strains, we included a sufficient genetic background for phenotypic variation in different selection traits. The inbreeding level which could affect the fertility traits like litter size (Holt et al. 2004) has increased during the long selection period in both environments (DUC 0.175 and DUhTP 0.592, respectively). But no abrupt rise of the average inbreeding level could be observed after changing to the new mouse facility with SPF hygienic standard. Thus, we suppose the heavier body mass of DUC_SPF_ mothers as a substantial factor or the increasing average number of born pups per litter. Those positive correlation between female body mass at mating and litter size were described by Holt et al. (2004). In addition, diets may have effects on reproductive performance including litter size (Ghosh et al. 2016) and an interaction between diet and genotype has been described in growth selected mice (Corva et al. 2004). Notably, diets with positive effects on body weight also increased reproductive performance in mice (Hoover-Plow et al. 1988), suggesting that the increases of body weight and litter size in DUC_SPF_ mice are connected. Also, in other mammalian species, dietary factors have been identified with an effect on litter size (Gado et al. 2016; Li et al. 2015). Under field conditions, a link between food quality and reproductive performance could not be observed in mice (Ylonen et al. 2003).

Probably due to increased litter size, DUC_SPF_ mice had lower body weights at birth than DUhTP_SPF_ mice. Between days 1 and days 21 after birth, DUC_SPF_ mice were characterized by significantly increased weight gain if compared to DUhTP_SPF_ mice resulting in higher body weights in DUC mice elder than 5 days if compared to DUhTP mice. The increased growth rate of DUC_SPF_ mice in the perinatal period may thus be due to accelerated compensatory growth or due to SPF husbandry. Very clearly, also in mice neonatal catch-up growth shortly after birth is associated with visceral obesity later in life (Isganaitis et al. 2009). Thus, reduced birth weight in fact may trigger catch-up growth and visceral obesity, and therefore contribute at least in part to the markedly increased body weights in DUC_SPF_ mice. However, in DUC_SPF_ mice also muscle weight was increased if compared to DUC_SB_ mice which is not explained by reduced birth weights or catch-up growth in the perinatal period because low birth weight was associated with increased fat mass but reduced lean mass in mice (Ju et al. 2016). Therefore, also an effect of maternal diet has to be considered which is well documented in the literature, e.g., by the postnatal control of growth or litter performance in suckling pigs (He et al. 2016). Finally, litter size is also a function of fetal mortality (Falconer 1960) and reproductive performance. In rats, the absence of pathogens under SPF conditions significantly increased litter size if compared to a conventional barrier, which was discussed in a context of higher numbers of corpora lutea and decreased post implantation losses of embryos or fetuses (Wakafuji et al. 1984).

### Effects of cryopreservation

For the efficient resettlement and establishment of the new and empty mouse facility, embryos from DUC_SB_ mice were cryoconserved by vitrification and transferred into pseudopregnant Crl:CD1(ICR) fosters defined by the SPF status. These generated DUC_SPF_ mice were used for initial stocking of the new SPF facility in Dummerstorf. Additionally, fresh embryos from line DUC_SB_ were transferred to pseudopregnant CD1 and DUC_SFP_ foster mothers. By contrast, fresh embryos from DUhTP_SB_ mice were used for transfer without cryopreservation. Therefore, we cannot exclude that cryoconservation in DUC_SPF_ mice is related to differential phenotype effects of SPF husbandry. In fact, higher body weights have been observed in cryopreserved mice if compared to controls (Dulioust et al. 1995). However, the increases in body weight were described as a long-term effect in adult mice only (Dulioust et al. 1995). Because growth activities were evident early after birth in DUC_SPF_ mice, cryopreservation seems not to account for the differential growth response of DUC versus DUhTP mice during SPF husbandry. Cryopreservation in human embryos explicitly did not affect growth during infancy and early childhood (Wennerholm et al. 1998) also not arguing for an effect of cryoconservation in young DUC_SPF_ mice. In general, it can be doubted that potential epigenetic modification established during handling of the embryo is transmitted to the offspring at all, because in cloned mice altered growth and obesity were found only in the born animal but not in the offspring of cloned mice (Tamashiro et al. 2002). In DUC_SPF_ mice, the phenotype of elevated growth is observed over 15 generations now, not supporting reversible effects of epigenetic modifications.

### Effects on running capacity

Animal husbandry in SPF conditions increased absolute weights of body, muscle, and different fat depots in DUC but not in DUhTP mice. These specific effects were not reflected by differential effects on running capacities in DUhTP mice versus DUC mice. Instead, SPF husbandry significantly elevated running capacities in both lines (*P* < 0.0001). Therefore, we may assume that the positive effects of husbandry on endurance exercise are related to improved animal health, due to the lack of specific pathogens and/or to the diet fed in the new SPF facility. Some pathogens (e.g., Helicobacter spp. or Pasteurellaceae) included in hygiene monitoring according to the FELASA guidelines (Mahler Convenor et al. 2014) are less clinically apparent. Under conditions of stress or in the presence of coinfections, clinical signs may become more prominent. Infections with mouse hepatitis virus (MHV) or the presence of endo- and ectoparasites severely can impact on animal health and result in life-threatening tissue damage during acute infection (Baker 2003). Because latent infections form various pathogens could be present under conditions of semi-barrier husbandry, it is conceivable that exercise capacity was limited in DUC_SB_ and DUhTP_SB_ mice by coinfection with different pathogens.

## Summary and conclusion

Here, we present a very rare direct comparison of different husbandry conditions on physiological parameters in animal models. The small number of comparative analyses, that can be found in the literature, relates to questions on health status in pigs (Clapperton et al. 2009), effects on immune state in mice (Beura et al. 2016), decontamination of mice (Wiese et al. 2007), or enzyme activity of alkaline phosphatase (Pickering and Pickering 1984). In the present comparative analysis, we demonstrate that both improved health and diet used under SPF conditions have multiple effects in the DUC/DUhTP mouse model. DUC_SPF_ mice have higher body weights at an age of more than 5 days and this effect is almost exclusively an effect of diet. The increased litter size might be an effect both of body weight and improved health and could be responsible for reduced birth weights in DUC_SPF_ mice. The strong increases of running capacities observed in both mouse lines could represent a benefit of improved health status in both mouse lines due to the lack of pathogens. However, very clearly we provide evidence that DUC and DUhTP mice differentially respond to environmental factors including improved health and an adjusted diet. From the differential increase or decrease of body fat, we may assume that energy metabolism is involved in the differential response of both mouse lines to environmental factors. Our results therefore strongly support the interaction of genomes with the environment, which may inspire the inclusion of environmental factors in phenotype analysis performed in animal models.
